# High-saturate-fat diet delays initiation of diethylnitrosamine-induced hepatocellular carcinoma

**DOI:** 10.1186/s12876-014-0195-9

**Published:** 2014-11-20

**Authors:** Xiao-Yan Duan, Qin Pan, Shi-Yan Yan, Wen-Jin Ding, Jian-Gao Fan, Liang Qiao

**Affiliations:** Department of Gastroenterology, Xinhua Hospital, Shanghai Jiaotong University School of medicine, Shanghai Key Laboratory of Children’s Digestion and Nutrition, Shanghai, 200092 China; Storr Liver Unit, Westmead Millennium Institute, the University of Sydney at the Westmead Hospital, Westmead, NSW 2145 Australia

**Keywords:** Nonalcoholic fatty liver disease, Diet, Hepatocellular carcinoma, Proliferation, Apoptosis

## Abstract

**Background:**

Nonalcoholic fatty liver disease (NAFLD) is a risk factor for hepatocellular carcinoma (HCC), but the association between a high-fat diet (HFD) and HCC is not fully understood. In this study, we investigated whether a high-saturate-fat diet affects hepatocarcinogenesis induced by administration of diethylnitrosamine (DEN).

**Methods:**

Adult SD rats were randomized into the following groups: normal chow diet (NCD), HFD, NCD + DEN, and HFD + DEN. The HFD contains 2% cholesterol and 10% lard oil. In mice with DEN treatment, the carcinogen was given via gavage. Mice were sacrificed at the end of 10, 12, and 14 weeks, respectively. The effects of HFD on hepatic carcinogenesis were assessed by HCC incidence, tumor differentiation, and the number and size of tumor nodules. Western blot and immunohistochemistry for proliferating cell nuclear antigen (PCNA), enzyme-linked immunosorbent assay (ELISA) for caspase-3, and real-time PCR for TNF-α and IL-6 further uncovered the proliferative and apoptotic properties of liver.

**Results:**

In contrast to the NCD group, DEN treatment (NCD + DEN group) led to hepatitis, cirrhosis, hepatic tumor, and decreased body weight. Interestingly, HFD, which induced hyperlipidemia and hepatic steatosis, attenuated DEN-related malnutrition and fibrosis progression in HFD + DEN group during 10–14 weeks. Moreover, the HFD + DEN group exhibited that the proportion of well differentiated HCC was much higher than that of NCD + DEN group. The number and average volume of HCC node were also significantly lowered in HFD + DEN group (*P* < 0.01-0.05). When compared to that of NCD + DEN group, there was an inhibited expression of PCNA, TNF-α, and IL-6, and activation of caspase-3 in the liver of HFD + DEN group at week 10 and 12.

**Conclusions:**

HFD restores malnutrition in the DEN-treated rats, which in turn inhibits the initiation of hepatic carcinogenesis and malignancy.

## Background

Hepatocellular carcinoma (HCC) is the fifth most common cancer and the third leading cause of cancer-related mortality worldwide [1,2]. HCC has clearly defined etiological factors such as chronic hepatitis B (CHB), chronic hepatitis C (CHC), long-term heavy alcohol consumption, exposure to aflatoxin, non-alcoholic steatohepatitis (NASH), malnutrition and obesity [3,4]. Clinical epidemiological and animal studies have shown that high-fat diets (HFD) with high-calorie intake significantly contribute to the development of obesity and NASH [5-9]. As HFD can induce lipid peroxidation and contribute to DNA damage, it is considered a risk factor for HCC [10-15].

However, some recent studies have interestingly suggested that HFD could delay the development of cancers in several organs such as breast, prostate, and liver [14-19]. Dietary intake of polyunsaturated fatty acid (PUFA)-enriched fish oil has been reported to limit post-operative metastasis and increase recurrence-free survival in melanoma-bearing rodents [14]. To further support an anticancer effect of fatty acids, it has been shown that n-3 PUFA supplements possess anti-tumorigenic and anti-migratory abilities in breast and prostate cancers [15-17]. Similarly, C57BL/6 DIO mice receiving fat forage demonstrate decreased HCC incidence and diminished area of hepatic foci [18]. HFD (contains 13.6-23.5% fat) inhibits the density, average area, and unit area of HCC foci in F344 rats [19].

To clarify the role of HFD during hepatocarcinogenensis, we have established an animal model of HCC using the classic hepatic carcinogen diethylinitrosamine (DEN) in rats chronically fed high-saturate-fat diet to experimentally recapitulate the development of liver cancer in the setting of fatty liver disease, and to understand the possible mechanisms therein.

## Methods

### Animals and experimental procedures

Adult male Sprague–Dawley rats (10 weeks old, average body weight 98.7 ± 6.3 g) (B&K Universal Group Ltd, Shanghai, China) were bred in a specific pathogen free animal unit of Shanghai Xinhua Hospital. Rats were randomized into four groups each treated with or without DEN (given **d**i**e**thyli**n**itrosamine 10 mg/kg/d by gavage) [20]. Animals were housed in plastic cages in groups of five and permitted *ad libitum* consumption of water and diet. Rats were allowed to acclimatize for a week on the normal chow diet (NCD) before grouping. Rats were given HFD (10% lard oil, 2% cholesterol, and 88% normal chow diet) [7] with or without DEN from week 2, and were closely monitored for physical abnormalities and were sacrificed at the end of weeks 10, 12, and 14. Serum samples were collected via cardiac puncture and maintained at −20°C until further analysis. Liver was quickly removed and weighted. Part of the liver tissues was snap frozen in liquid nitrogen for further analysis. Two small pieces (1 × 1 × 0.5 cm [3]) of liver tissues were immediately fixed in 10% neutral-buffered formalin for histological analysis. All animal studies were approved by the Shanghai Jiao Tong University Institutional Animal Care and Use Committee.

### Serum lipid profile and liver function tests

Serum level of total cholesterol (TC), triglyceride (TG), high-density lipoprotein cholesterol (HDL-C), low-density lipoprotein cholesterol (LDL-C), and albumin was measured by using a commercial kit (Wako Pure Chemical Industries, Richmond, VA, USA). Serum level of alanine aminotransferase (ALT) and aspartate aminotransferase (AST) was measured using multichannel automatic analyzer (Bayer Advia 1650, Leverkusen, Germany).

### Histologic analysis

Rat liver tissues were embedded in paraffin, and then cut into 4 μm thickness sections. Slides were subjected to routine hematoxylin-eosin (H&E) staining and Van-Greson (VG) staining. Liver fibrosis was graded according to the Metavir Score system, and chronic hepatitis activity index (HAI) proposed by Knodell was adopted to calculate the liver inflammatory activity score [21]. HAI = P + L + 2 × (PN + BN). P represents periportal inflammation, L represents lobular inflammation, PN represents piecemeal necrosis, and BN represents bridging and multi-lobular necrosis. Development of HCC was studied by two independent pathologists who were blind to the study.

### Measurement of cell proliferation and apoptosis

The ability of hepatocytes to undergo apoptosis in response to the above-mentioned treatment regimens was examined by measuring the expression levels of active form of Caspase 3 in liver tissues using commercial enzyme-linked immunosorbent assay (ELISA) kits (XiTang Biothch Inc., Shanghai, China) according to the manufacturer’s instructions.

Measurement of PCNA by Western blot: Total hepatic protein was prepared and quantified by the bicinchoninic acid method (Pierce, Rockford, IL, USA). Thirty micrograms of protein per sample was loaded onto a 10% SDS polyacrylamide gel. After electrophoresis, the protein was transferred onto a polyvinylidenedifluoride membrane (PVDF) (Millipore, Billerica, MA, USA). The membrane was incubated with anti-PCNA antibody (1:1500, Epitomics, USA) for overnight at 4°C and then with HRP-conjugated goat anti-mouse IgG (1:4000; Jackson ImmunoResearch Laboratories Inc, West Grove, PA, USA) for 2 h at room temperature. Following three washes with TBST, the signal on the membrane was developed by using SuperSignal West Pico Chemiluminescent Substrate (Pierce, Rockford, USA). β-actin was used as a loading control (Santa Cruz, Santa Cruz, CA, USA) (1:500).

Measurement of PCNA by immunohistochemistry: Sections (5 μm thick) were cut from formalin-fixed and paraffin embedded liver samples. After a standard dehydration-rehydration procedure, liver sections were incubated with 3% H_2_O_2_ for 10 min to quench endogenous peroxidase activity. The sections were then heated using a steamer for 20 min in 10 mM sodium citrate (pH 6.0) buffer to retrieve antigen. The routine biotin-streptavidin immunohistochemical method consisted of sequential incubations in goat serum blocking solution, monoclonal anti-PCNA (1:100, Epitomics, USA) biotinylated goat anti-rabbit IgG. The liver specimens were finally treated with diaminobenzidine substrate and then counterstained with hematoxylin.

### Real-time PCR

RNA isolation and purification: Total RNA was isolated from frozen liver tissues using Trizol reagent. (1) Reverse transcription: cDNA was synthesized from the isolated RNA by using RevertAid™ First Strand cDNA Synthesis Kit (Fermentas, Lithuania). (3) Real-time PCR: Gene-specific primer sequences were designed using the Primer Premier 5.0 software and custom-synthesized by Shanghai Sangon Biological Engineering Technology and Service Co. Ltd. (China). Glyceraldehyde 3-phosphate dehydrogenase (GAPDH) gene was used as an internal control. The primer sequences utilized were as follows:

- GAPDH: sense 5′-TGATGGGTTTCCCATTGATGA-3′,
- anti-sense 5′-TGATTCTACCCACGGCAAGTT-3′;
- IL-6: sense 5′-TCAATGAGGAGACTTGCCTG-3′,
- anti-sense 5′-GATGAGTTGTCATGTCCTGC-3′
- TNF-α: sense 5′-CTTCTGCCTGCTCTTTGGA 3′,
- anti-sense 5′-AGGAACAGCTGGCTGCCTGTCT 3′.

The PCR reaction was carried out in each well using 20 μL reaction mixture containing 10 μL SYBR Premix Ex Taq, 0.4 μL primer mix (including forward and reverse primers) and 1 μL cDNA diluted in Rnase-free water. The ∆∆CT method was used for relative quantification of the results.

### Statistics

Data were expressed as mean ± standard deviation (SD). SPSS 16.0 statistical package was used to conduct the statistic analysis. One way ANOVA was used for comparing the grading scores of different groups, and Fisher’s exact test was used for rate comparison. A *P* value of less than 0.05 (two-tailed) was considered statistically significant.

## Results

### Effect of HFD and DEN on nutrition status

In rats fed NCD, administration of DEN (i.e., NCD + DEN group) led to a significant weight loss and a marked decrease in BMI at weeks 10, 12, and 14 (*P* < 0.01, compared to the NCD alone group, Table 1). In sharp contrast, when the rats fed on HFD were exposed to DEN (i.e., HFD + DEN group) for the same duration, there was a less reduction in body weight and BMI (*P* < 0.01, Table 1). Despite the improved nutritional indexes, body weight, body length and BMI of the HFD + DEN group were still lower than those of the HFD group (*P* < 0.01, Table 1).

**Table 1:** **Effect of high-saturate-fat diet and DEN on nutrition status, serum lipid profile and liver function test**

**Week**	**Group**	**n**	**Body weight (g)**	**Body length (cm)**	**BMI (g/cm** ^2^ **)**	**TC (mmol/L)**	**TG (mmol/L)**	**HDL-C (mmol/L)**	**LDL-C (mmol/L)**	**ALT (U/L)**	**AST (U/L)**	**ALB (g/L)**
10	NCD	8	379.00 ± 27.83	24.13 ± 0.64	0.62 ± 0.05	1.47 ± 0.17	0.17 ± 0.05	1.01 ± 0.13	0.89 ± 0.09	42.38 ± 7.76	133.75 ± 22.30	35.24 ± 1.13
	HFD	8	486.88 ± 34.50	26.55 ± 0.69	0.66 ± 0.03	2.13 ± 0.50	0.38 ± 0.34	1.45 ± 0.25^#^	1.39 ± 0.36^#^	48.38 ± 14.29	172.63 ± 59.49	36.18 ± 2.20
	NCD + DEN	7	276.9 ± 40.8^^&^	23.79 ± 1.19	0.49 ± 0.05^^^&&^	2.26 ± 0.37^^^	0.47 ± 0.18^^^	1.17 ± 0.35	1.32 ± 0.25^	170.01 ± 77.55^^&^	364.17 ± 261.10	36.16 ± 2.34
	HFD + DEN	7	363.13 ± 21.91^▲▲^^$$^	25.14 ± 0.75^▲▲^	0.57 ± 0.03^▲▲$$^	4.05 ± 1.41^▲^	0.54 ± 0.22	2.27 ± 0.48^▲▲^**	2.51 ± 1.00^▲^**	144.90 ± 22.83**^$$^	187.74 ± 17.89	38.96 ± 1.29^▲^*^$^
12	NCD	8	429.50 ± 19.32	25.81 ± 0.70	0.65 ± 0.03	1.58 ± 0.14	0.35 ± 0.15	1.13 ± 0.15	1.02 ± 0.10	36.63 ± 18.12	131.75 ± 28.74	37.25 ± 2.31
	HFD	8	531.63 ± 36.62	27.53 ± 0.44	0.70 ± 0.04	2.52 ± 0.55^#^	0.30 ± 0.11	1.82 ± 0.30^##^	1.76 ± 0.42^#^	166.38 ± 72.26^##^	235.00 ± 58.89^##^	36.95 ± 2.59
	NCD + DEN	7	289.9 ± 65.6^^^&&^	23.71 ± 2.06	0.51 ± 0.04^^^&&^	2.55 ± 0.40^^^	0.46 ± 0.13	1.32 ± 0.15	1.54 ± 0.18^	118.64 ± 20.55^^^^	258.71 ± 48.22^^^^	34.57 ± 3.31
	HFD + DEN	7	384.3 ± 28.9^▲▲$$^	25.43 ± 0.53	0.59 ± 0.04^▲▲$$^	4.64 ± 0.79^▲▲^**	0.59 ± 0.14*$	2.77 ± 0.23^▲▲^**	2.88 ± 0.56^▲▲^**	149.69 ± 35.13**	272.72 ± 102.56**	38.90 ± 1.17^▲^
14	NCD	8	442.20 ± 49.66	26.00 ± 0.91	0.66 ± 0.08	1.75 ± 0.16	0.28 ± 0.18	1.12 ± 0.16	1.09 ± 0.10	53.00 ± 9.62	148.56 ± 27.05	37.15 ± 1.71
	HFD	8	571.1 ± 36.58^##^	27.25 ± 0.75^#^	0.77 ± 0.03^#^	2.90 ± 0.63^#^	0.39 ± 0.14	1.93 ± 0.31^##^	2.62 ± 0.63^##^	139.10 ± 63.72^#^	260.00 ± 90.15^#^	36.90 ± 0.91
	NCD + DEN	9	285.72 ± 53.95^^^&&^	23.94 ± 1.51^^^^	0.50 ± 0.05^^^&&^	3.16 ± 0.63^^^^	0.63 ± 0.23^^^	1.61 ± 0.36	1.77 ± 0.37^^^&^	162.03 ± 34.72^^^^	365.11 ± 70.68^^^^	36.89 ± 3.07
	HFD + DEN	10	386.26 ± 47.71^▲▲^^$$^	25.75 ± 0.98^▲▲^	0.58 ± 0.04^▲▲$$^	4.85 ± 0.95^▲▲^**^$$^	0.56 ± 0.20*	2.65 ± 0.25^▲▲^**^$^	3.04 ± 0.69^▲▲^**	151.86 ± 23.54**	248.90 ± 68.52^▲▲^*	40.00 ± 1.96^▲^*^$^

**NCD: n**ormal **c**how **d**iet; **HFD: h**igh saturate **f**at **d**iet; **DEN**: **d**i**e**thyli**n**itrosamine; TC, total cholesterol; TG, triglyceride; HDL-C, high-density lipoprotein cholesterol; LDL-C, low-density lipoprotein cholesterol; ALT, alanine aminotransferase; AST, aspartate aminotransferase; ALB, albumin.

HFD + DEN *vs* NCD + DEN: ^▲^ *P* < 0.05, ^▲▲^ *P* < 0.01; HFD + DEN *vs* NCD: *P < 0.05, **P < 0.01; HFD + DEN *vs* HFD: ^$^ *P* < 0.05, ^$$^ *P* < 0.01; NCD + DEN *vs* NCD: ^^^ *P* < 0.05, ^^^^ *P* < 0.01; NCD + DEN *vs* HFD: ^&^ *P* < 0.05, ^&&^ *P* < 0.01; HFD *vs* NCD: ^#^ *P* < 0.05, ^##^ *P* < 0.01.

### High-saturate-fat diet improved liver function test in DEN-treated rats

As shown in Table 1, NCD fed rats displayed a significant elevation in serum ALT and AST following DEN treatment (i.e., the NCD + DEN group *vs* the NCD along group). Simultaneous treatment with HFD and DEN (the HFD + DEN group), however, dramatically reduced the AST level at the end of 14 weeks (*P* <0.01 compared to the NCD + DEN group, Table 1). In parallel, there was an increased serum concentration of ALB in the HFD + DEN group at the weeks 10 to 14 (*P* < 0.05 compared to the NCD + DEN group, Table 1).

### High-saturate-fat diet induced hyperlipidemia and hepatic steatosis

Compared to rats in the NCD + DEN group, rats in the HFD + DEN group demonstrated significant elevation of serum TC and LDL-C (Table 1). In contrast, there was no significant difference in serum level of TG between the NCD + DEN and the HFD + DEN groups.

**Figure 1:**
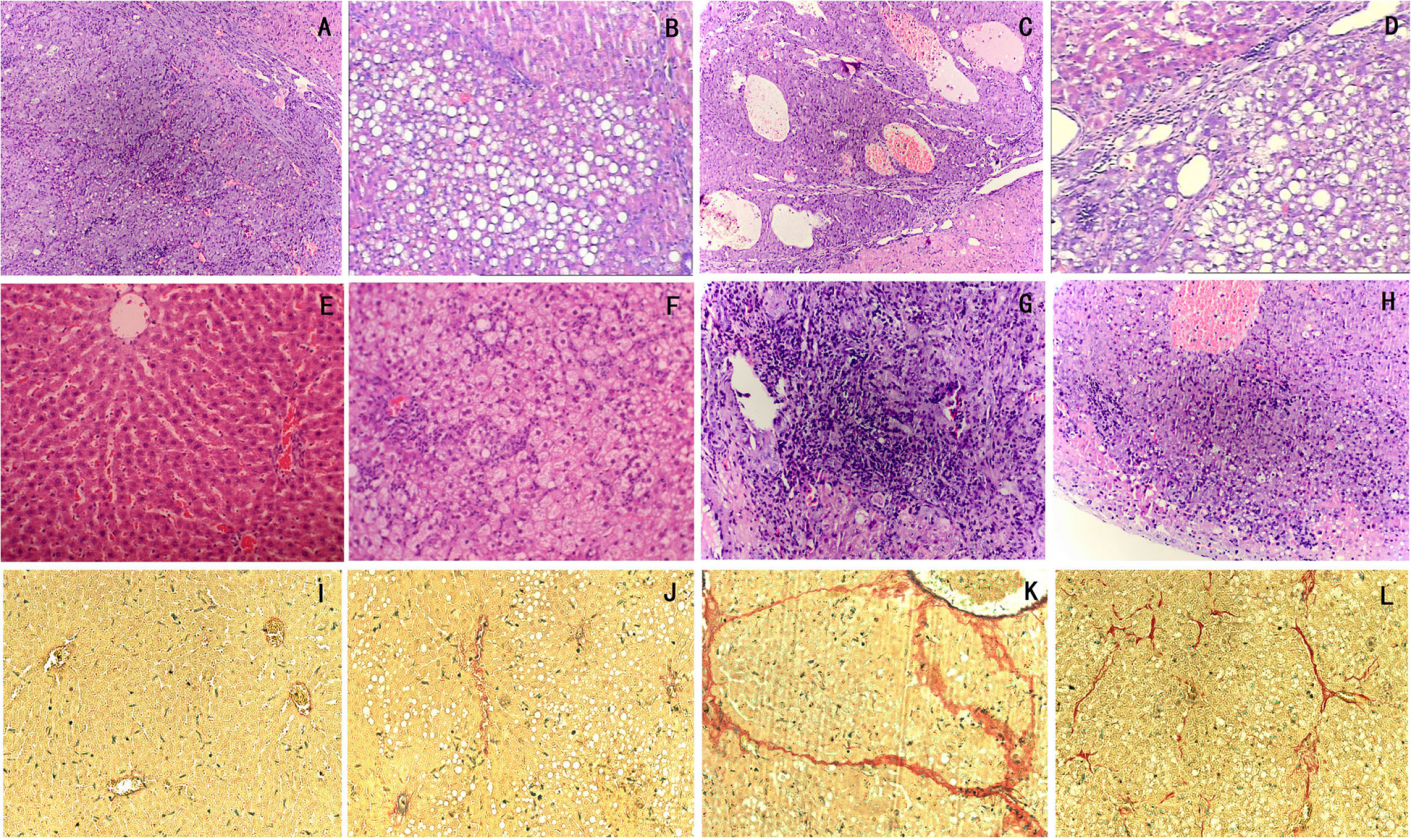
**Histological features by H&E and VG staining.** Liver tissues of H&E staining displayed the well differentiated HCC (**A**, 10w) and middle differentiated HCC (**B**, 12w) in DEN + CD group, hepatic steatosis (**C**, 10w) and well differentiated HCC with steatosis (**D**, 12w) in DEN + HFD group. Then H&E staining was performed in liver tissues of NCD (**E**, n = 8), HFD (**F**, n = 8), NCD + DEN (**G**, n = 9), and HFD + DEN (**H**, n = 10) groups after 14 weeks. Liver tissues of VG staining were obtained from rats fed on NCD (**I**, n = 8), HFD (**J**, n = 8), NCD + DEN (**K**, n = 7), and HFD + DEN (**L**, n = 7) for 10 weeks. Magnification: ×200.

In accordance with the above changes, HFD fed rats (including the HFD and HFD + DEN groups) displayed moderate to severe hepatic steatosis, hepatocyte ballooning, and infiltration of lymphocytes and mononuclear cells within lobule and portal area (Figure 1). In contrast, the livers in the rats of the NCD + DEN group showed moderate to severe hepatic necroinflammation without or only with mild steatosis.

### High-saturate-fat diet delayed DEN-related liver fibrosis and HCC

**Figure 2:**
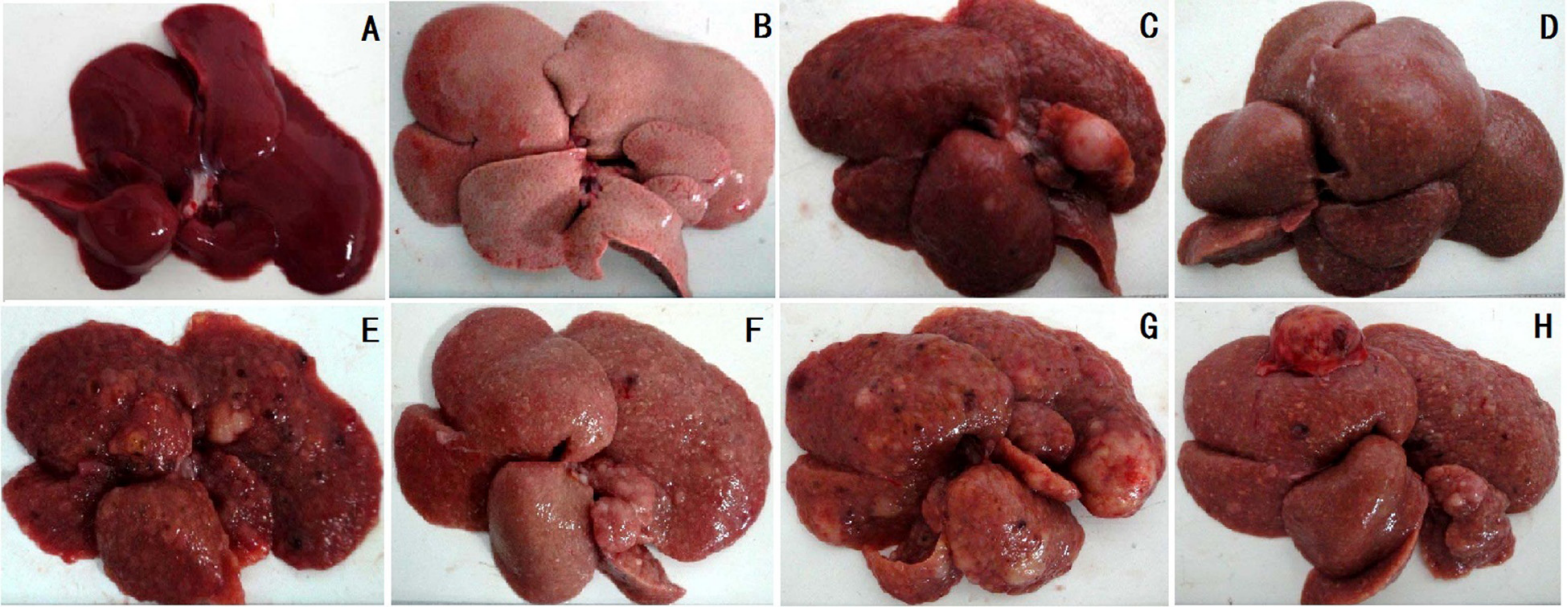
**Gross specimen of rat liver.** Liver specimen from NCD (**A**, 14 weeks (n = 8) ), HFD (**B**, 14 weeks (n = 8)), NCD + DEN (**C**, 10 weeks (n = 7); **E**, 12 weeks (n = 7); **G**, 14 weeks (n = 9)) and HFD + DEN groups (**D**, 10 weeks (n = 7); **F**, 12 weeks (n = 7); **H**, 14 weeks (n = 10)) displays the effect of DEN and/or HFD on carcinogenesis.

Subsequent pathological disorders, including hepatic necroinflammation, nodular regeneration, liver fibrosis/cirrhosis, dysplasia and HCC could be observed in rats with DEN administration (Figures 1 and 2). No rats developed obvious pathological changes in the NCD group. In the HFD group, Grade 1 liver fibrosis was observed in 12.5% of rats by week 12 and 25% by week 14. When the NCD fed rats were given DEN (i.e., NCD + DEN group), all rats developed Grade 4 fibrosis by weeks 10–14. In contrast, in the HFD + DEN group, there was a delayed fibrogenesis in the liver with slightly reduced HAI (Table 2), in that Grade 4 fibrosis only developed in 57.1% of rats by week 10, 71.4% by week 12, and 90% by week 14 (Table 2).

**Table 2:** **High-saturate-fat diet delayed DEN-related liver fibrosis**

**Week**	**Group**	**n**	**HAI**	**Liver fibrosis**				
				**0 (%)**	**1 (%)**	**2 (%)**	**3 (%)**	**4 (%)**
10	NCD	8	0	8 (100)	0 (0)	0 (0)	0 (0)	0 (0)
	HFD	8	2.5 ± 1.0	8 (100)	0 (0)	0 (0)	0 (0)	0 (0)
	NCD + DEN	7	12.1 ± 2.2	0 (0)	0 (0)	0 (0)	0 (0)	7 (100)
	HFD + DEN	7	10.7 ± 2.9	0 (0)	0 (0)	1 (14.3)	2 (28.6)	4 (57.1)
12	NCD	8	0	8 (100)	0 (0)	0 (0)	0 (0)	0 (0)
	HFD	8	3.1 ± 1.2	7 (87.5)	1 (12.5)	0 (0)	0 (0)	0 (0)
	NCD + DEN	7	14.9 ± 4.5	0 (0)	0 (0)	0 (0)	0 (0)	7 (100)
	HFD + DEN	7	11.0 ± 1.2	0 (0)	0 (0)	0 (0)	2 (28.6)	5 (71.4)
14	NCD	8	0	8 (0)	0 (0)	0 (0)	0 (0)	0 (0)
	HFD	8	3.6 ± 1.9	6 (75.0)	2 (25.0)	0 (0)	0 (0)	0 (0)
	NCD + DEN	9	15.1 ± 3.1	0 (0)	0 (0)	0 (0)	0 (0)	9 (100)
	HFD + DEN	10	14.0 ± 2.3	0 (0)	0 (0)	0 (0)	1 (10)	9 (90)

**NCD, n**ormal **c**how **d**iet; **HFD, h**igh saturate **f**at **d**iet; **DEN, d**i**e**thyli**n**itrosamine; HAI, hepatitis activity index.

Following DEN exposure, there was a marked increase in the incidence of hepatic tumors, most of which were HCC, with the only one case of mixed HCC and cholangiocarcinoma in the NCD + DEN group at week 12. Compared to the NCD + DEN group, rats in HFD + DEN group developed less hepatic tumor nodules by weeks 10 (0% *vs* 42.9%) and week 12 (28.6% *vs* 71.4%).

Further analysis revealed that rats in the HFD + DEN group developed less number of the tumor nodules compared to rats in the NCD + DEN group by week 14 (Table 3), and the number of rats with large nodules (≥5 mm) was smaller in the HFD + DEN group compared to the NCD + DEN group (*P* < 0.05) (Table 3). The average volume of tumor nodules was also significantly smaller in the HFD + DEN group than in the NCD + DEN group (*p* < 0.01) (Table 3). Additionally, the hepatic tumours in the HFD + DEN group were generally better differentiated than tumours in the NCD + DEN group at week 14 (Table 3).

**Table 3:** **High-saturate-fat diet delayed DEN-induced HCC formation (week 14)**

**Group**	**Differentiation**		**Total no. of tumour nodules/rat**	**No. of rats with tumour nodules ≥5 mm**	**Average tumour nodules (mm** ^**3**^ **)**
	**High**	**Low**			
NCD + DEN	1	8	15.33 ± 6.32	2.33 ± 1.32	3196.86 ± 7772.85
HFD + DEN	4	6	7.80 ± 4.37**	1.20 ± 0.79*	513.34 ± 1132.90**

**NCD: n**ormal **c**how **d**iet; **HFD: h**igh saturate **f**at **d**iet; **DEN**: **d**i**e**thyli**n**itrosamine.

HFD + DEN *vs* NCD + DEN: *P < 0.05, **P < 0.01.

### Anti-proliferative and pro-apoptotic effect of HFD

**Figure 3:**
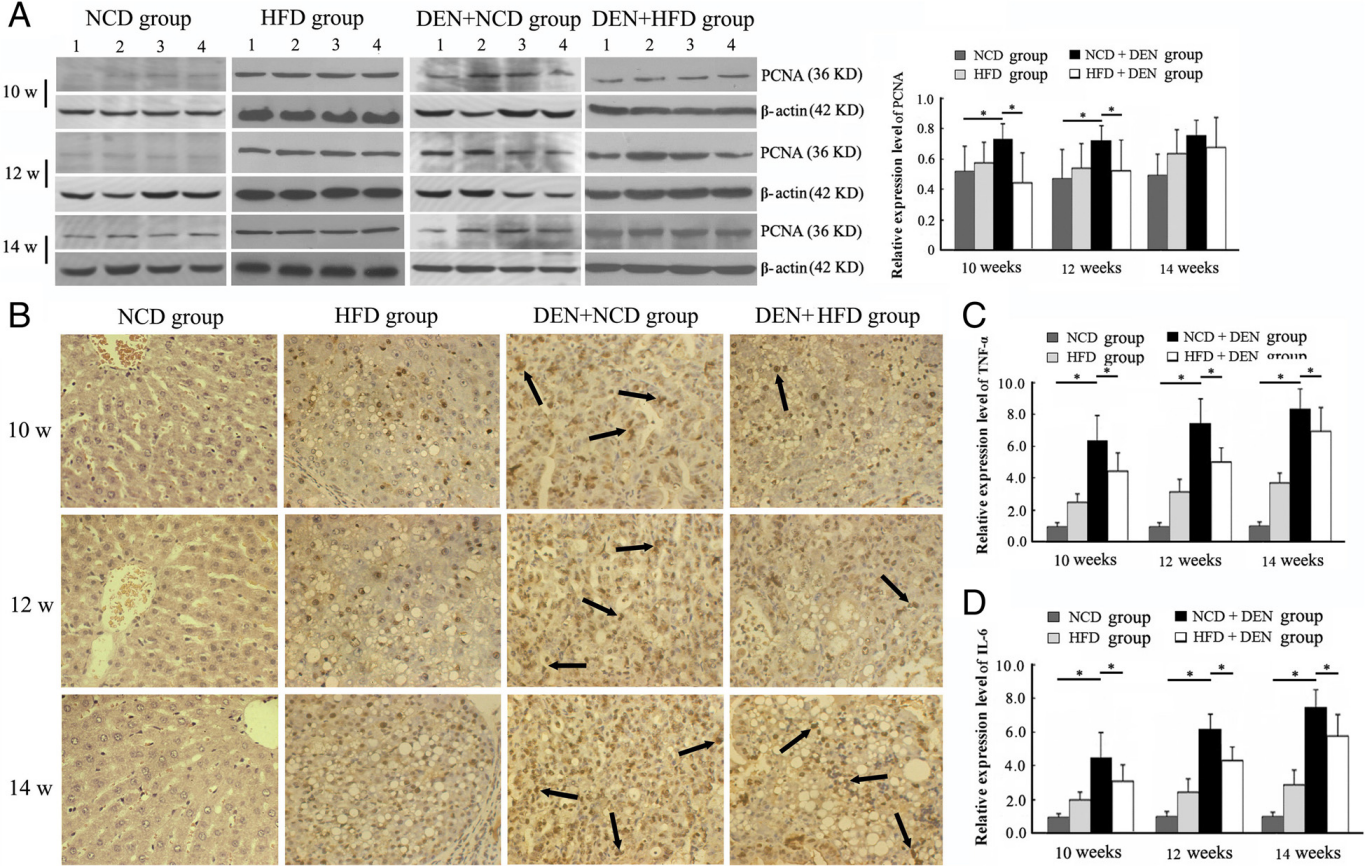
**HFD reduces the hepatic expression of PCNA, TNF-α, and IL-6. (A, B)** Hepatic expression of PCNA by Western blot **(A)** and immunohistochemistry **(B)**. Compared to rats from NCD + DEN group (10w: n = 7, 12w: n = 7, 14w: n = 9), rats from the HFD + DEN group (10w: n = 7, 12w: n = 7, 14w: n = 10) exhibited marked decrease in the expression of PCNA, particularly at weeks 10 and 12. Arrows indicate the liver cells positive for PCNA. Magnification: ×400. Hepatic expression of TNF-α **(C)**, and IL-6 **(D)** by real-time PCR. *P <0.05.

As demonstrated by Western blot, the expression level of PCNA in the liver at week 14 was similar between the NCD + DEN group and the HFD + DEN group. However, there was a more significant reduction in the PCNA expression in HFD + DEN group by weeks 10 and 12 compared to rats in the NCD + DEN group of the same respective time points (Figure 3A). The effect of HFD on HCC proliferation was further confirmed by immunohistochemically staining for PCNA. As compared to that in the DEN + NCD group (10 week: 55.71 ± 8.28 cells/field; 12 week: 56.57 ± 10.26 cells/field; 14 week: 62.11 ± 8.45 cells/field), PCNA-positive HCC cells in the DEN + HFD group were much less (10 week: 34.29 ± 5.94 cells/field; 12 week: 41.14 ± 7.06 cells/field; 14 week: 60.50 ± 11.68 cells/field) at the time points of 10 and 12 week (*P* < 0.05, Figure 3B).

**Figure 4:**
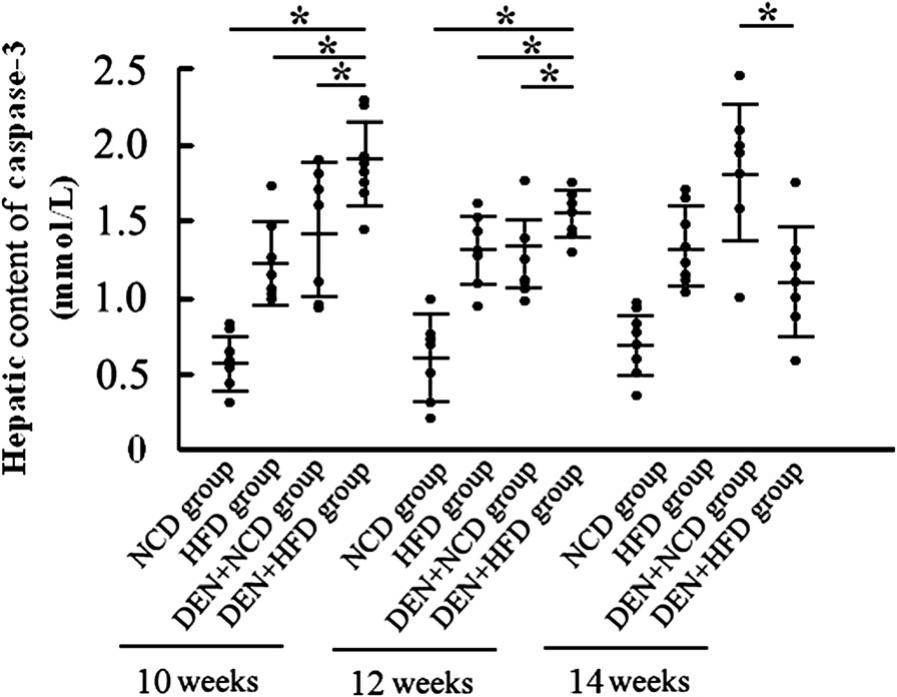
**HFD predisposes rat to hepatic apoptosis.** Hepatic level of caspase-3 was determined by ELISA. Increased hepatic level of caspase-3 was found in the HFD + DEN group (10w: n = 7, 12w: n = 7, 14w: n = 10) than in the NCD + DEN groups (10w: n = 7, 12w: n = 7, 14w: n = 9). *: *P* <0.05.

We also measured the hepatic content of Caspase-3 as a marker of apoptosis by ELISA. At weeks 10 and 12, there was a significantly increase in the hepatic level of Caspase-3 in the HFD + DEN group compared to the NCD + DEN group (*P* < 0.05). However, the hepatic Caspase-3 content of the DEN + HFD group was significantly lower than that of the DEN + NCD group at the time point of 14 week (*P* <0.05) (Figure 4).

### Effect of HFD on TNF-α and IL-6 expression

When compared to that of NCD group, rats of the DEN + NCD group were suffered from the significant increase of TNF-α and IL-6 mRNA levels in the liver at time points of 10, 12, and 14 week (*P* < 0.05, Figure 3C-D). Contrastively, translational level of these inflammatory cytokines experienced statistical inhibition after the exposure to HFD (DEN + HFD group vs DEN + NCD group, *P* < 0.05, Figure 3C-D).

## Discussion

Malnutrition and ensuing weigh loss reflect the most common complications of cancer patients and also associated with some malignant tumor [22-24]. In response to elevated energy consumption in cancer patients, more calories than normal are needed to maintain the nutrition status and organ function [22]. Recently study has revealed that low-fat intake could lead to the deterioration of energy status in HCC patients, and this was associated with a poor recovery from invasive treatments [25]. Consistently, a fat-enriched artificial liquid diet (20 non-protein kcal/kg per day) has been reported to restore the weight loss in patients with gastrointestinal carcinomas [23]. HFD with ketogenic regimen, in which up to 80% of the energy is supplied by medium chain triglycerides (MCT), also induces the weight gain in mice with colon adenocarcinoma [24]. HFD, therefore, is suggested to normalize the nutrition status against cancer-related cachexia.

In our animal study, we observed that rats treated with NCD + DEN exhibited weight loss, delayed growth, decreased BMI, and a derangement of liver function. In contrast, rats treated with HFD + DEN showed increased body weight, body length and BMI. This nutritional improvement was accompanied by partial improvement of serum aminotransferases and albumin. Additionally, in HFD fed rats, there was an increased serum level of TC, LDL-C and HDL-C. Thus, HFD may be protective against DEN-related malnutrition and liver dysfunction test.

Recent studies have indicated that nutrition status, as defined by such parameters as serum TC, HDL-C, and LDL-C, has profound impact on the initiation and progression of hepatic carcinogenesis [18,19]. There is a significant inverse correlation between serum HDL-C level and the risk of cancer, which is independent of age, sex, BMI, diabetes, LDL-C, and smoking history [26]. On the other hand, HCC patients show lower levels of serum TC, HDL-C, LDL-C, and triglycerides compared to patients with CHC and controls [27,28]. Low serum level of LDL-C even serves as a significant and independent predictor of HCC in patients with CHB or CHC [29-31]. Additionally, high serum level of TC was negatively associated with the development of liver cancer in both sexes [32].

Similar results could be observed in our experiments that the incidence rate of HCC was lower in the HFD + DEN group, which was characterized by increased serum levels of TC, HDL-C and LDL-C, in comparison with that of the DEN + NCD group at weeks 10 and 12. Afterward, all rats of both the NCD + DEN group and the HFD + DEN group developed HCC at week 14. However, the total number of tumour nodules, the proportion of large tumour nodules (≥5 mm), and the average volume of tumour nodules were significantly less in the HFD + DEN group than those in the NCD + DEN group. Furthermore, the tumours in the HFD + DEN group showed better differentiation status than those in the NCD + DEN group. Therefore, HFD appeared to attenuate the occurrence of HCC and malignant differentiation in rat HCC model induced by DEN.

Although saturated fatty acid and unsaturated fatty acids share the nutritional role in tumour-bearing animals, they differ from each other in their roles in carcinogenesis [14,15,19,33]. In a DEN-related rodent model of HCC, high-saturated-fat diet (containing 48% of calories as palm oil) but not the high-polyunsaturated-fat diet (containing 48% of calories as safflower oil) reduced the occurrence of γ-glutamyl transpeptidase-positive and ATPase-negative foci in the liver [33]. When compared to those in corn-oil-diet treated rats, the density, average area and unit area of HCC foci are also inhibited in the male Fischer 344 rats after their exposure to 13.6% and 23.5% lard diets [34]. In consistent to the short-term effect of fatty acids, 100% of mice with a diet of high polyunsaturated-to-saturated fatty acid (P/S) ratio develop lymphoma at 12 to 14 months. In contrast, only 70% of mice fed low P/S diet developed lymphoma [19]. Thus, saturated fatty acid, but not unsaturated fatty acids, might be able to inhibit the initiation of carcinogenesis.

Treatment of DEN usually gives rise to stepwise histological appearances of hepatitis, liver fibrosis, cirrhosis, hepatocellular adenoma and HCC [35-38]. However, combination of high-saturated-fat diet and DEN seemed to ameliorate hepatic necroinflammation and liver fibrogenesis with reduced serum aminotransperases in this study. As compared to the rats in the NCD + DEN group, rats in the HFD + DEN group showed a delayed progression in liver fibrosis and cirrhosis with reduced HAI at the end of 10, 12, and even 14 weeks.

The impact of HFD on the development of liver fibrosis and HCC formation may be related to the inhibitory effect of HFD on cell proliferation and induction of apoptosis [15,39]. *In vitro* studies have shown that cholesterol deficiency in the culture medium could promote the neoplastic cellular growth [40,41]. In our study, rats in the HFD + DEN group exhibited significantly reduced proliferation of hepatocytes (expression of hepatic PCNA by Western blot and immunohistochemistry) than those in the NCD + DEN group at the end of 10 and 12 weeks. These data support an inhibitory role of high-saturated-fat and 2% cholesterol diet in the initiation of HCC. However, there is no significant difference of hepatocytes proliferation between the DEN + HFD group and DEN + NCD group after 14 weeks.

Resistance to apoptosis represents a fundamental feature of HCC. Activation of hepatic caspase-3, which serves as an indispensible executor of apoptosis pathway [42], was found to be more prominent in the rats of the HFD + DEN group than in the NCD + DEN group, indicating a more active apoptotic state in rats of the HFD + DEN group. Activation of caspase-3 would in turn activate the mitochondrial apoptosis pathway and predispose HCC to programmed cell death [43,44]. An inhibitory effect on the production of hepatic TNF-α and IL-6, which take the critical place in carcinogenesis [45], may shed light on the mechanisms of HFD treatment that delays the DEN-induced HCC in its early stage.

Our study has a few limitations. First, we did not include rats fed HFD with similar daily calorie intake to the NCD group in this study. Thus we could not definitely conclusive that HFD inhibits the initiation of DEN-induced hepatocarcinogenesis, and the effect of HFD in this study might partly depend on total calorie intake rather than fat intake.

## Conclusions

Long-term high-saturated-fat diet with increased total calorie intake facilitates the normalization of the DEN-induced malnutrition in SD rats. This nutritional improvement attenuates histological activity of necroinflammatory and liver fibrosis progression. Moreover, it causes lower number and average volume of HCC node in the rats as compared to the control, possibly through the anti-proliferative and pro-apoptotic effect of HFD. High-saturate fat diet and high calorie intake, therefore, may serve as an inhibitor of the initiation of hepatic carcinogenesis and malignant progression in a rat DEN model.
